# Treatment of Cholera

**Published:** 1885-01

**Authors:** 


### TREATMENT OF CHOLERA.

In view of the expected visit of the cholera to this country during the coming year, any contribution to medical literature, bearing upon the treatment of this disease, should receive careful and earnest consideration on the part of the medical profession.

By the researches of Dr. Koch, it is now known that acids are most useful to kill the cholera microbe, and have been successfully employed by the profession in Europe.

Dr. Chas. Gatchell, of Chicago, in his "Treatment of Cholera," says: "As it is known that the cholera microbe does not flourish in acid solutions, it would be well to slightly acidulate the drinking water. This may be done by adding to each glass of water half a teaspoonful of Horsford's Acid Phosphate. This will not only render the water of an acid reaction, but also render boiled water more agreeable to the taste. It may be sweetened if desired. The Acid Phosphate, taken as recommended, will also tend to invigorate the system and correct debility, thus giving increased power of resistance to disease. It is the acid of the system, a product of the gastric functions, and hence will not create that disturbance liable to follow the use of mineral acids."

The following case is reported from Bangkok, Siam, and may be relied on as authentic. About three months ago a native was attacked with cholera. An American missionary attended him, and administered all medicines he could, but at last the man was so far gone that they gave up all hopes of recovery, and would do no more. Relatives of the patient begging the doctor not to give him up as lost, the doctor thought of Horsford's Acid Phosphate. After the second dose the patient commenced to revive, and in six hours after he was pronounced out of danger.

